# Effects of research tool patents on biotechnology innovation in a developing country: A case study of South Korea

**DOI:** 10.1186/1472-6750-9-25

**Published:** 2009-03-26

**Authors:** Kyung-Nam Kang, Tae-Kyu Ryu, Yoon-Sik Lee

**Affiliations:** 1 Korea Institute of Intellectual Property, KIPS center 16th floor, 647-9, Seoul 135-980, Republic of Korea; 2 Technology Management, Economics and Policy Program, Seoul National University, Seoul 151-744, Republic of Korea; 3 School of Chemical and Biological Engineering, Seoul National University, Seoul 151-744, Republic of Korea

## Abstract

**Background:**

Concerns have recently been raised about the negative effects of patents on innovation. In this study, the effects of patents on innovations in the Korean biotech SMEs (small and medium-sized entrepreneurs) were examined using survey data and statistical analysis.

**Results:**

The survey results of this study provided some evidence that restricted access problems have occurred even though their frequency was not high. Statistical analysis revealed that difficulties in accessing patented research tools were not negatively correlated with the level of innovation performance and attitudes toward the patent system.

**Conclusion:**

On the basis of the results of this investigation in combination with those of previous studies, we concluded that although restricted access problems have occurred, this has not yet deterred innovation in Korea. However, potential problems do exist, and the effects of restricted access should be constantly scrutinized.

## Background

In a knowledge-based economy, it is assumed that the patent system is an effective incentive mechanism for research and development (R&D), particularly in fields such as biotechnology where innovations have long gestation periods. However, the patent system is an imperfect mechanism because privatization can mitigate these benefits [1]. Recent studies have suggested that too much patenting could potentially deter innovation [1-6]. Concerns about over-patenting and its negative effects are widespread [2], which has prompted researchers to investigate the effects of patents [7-17]. Many of these studies have focused on the field of biotechnology in particular [7-13]. The "upstream" patents in this field have enormous power because inventions cannot be invented around (see Note A) and are of crucial importance to researchers [10].

Heller and Eisenberg argued that biomedical innovation has become susceptible to a so called "tragedy of the anticommons," which can emerge when each of the multiple owners of innovations has a right to exclude others from a scarce resource [1,7]. Under these circumstances, transaction costs become too high to collect all the relevant information for further research, which results in an under use of patented biotechnological information [1,2]. Shapiro also raised similar concerns, where he referred to this phenomenon as the "patent thicket." He argued that technologies that depended on the agreement of multiple parties were susceptible to delay by any one member [6,7]. Indeed, there has been some evidence that broad foundational patents can "block" research pathways [7]. In addition, it may stifle or misdirect research and retard the development of socially beneficial products and processes [2].

Empirical investigations in the US have also confirmed the existence of access problems, especially for upstream discoveries. Cho et al. conducted a survey of clinical laboratory directors who performed DNA-based genetic tests to examine the potential effects of patents. The respondents reported that their perceptions of the effects of patents on the cost, access, and development of genetic tests or data sharing among researchers were negative [8]. In addition, Thumm's survey, conducted in Switzerland, also confirmed that the concepts of anticommons, patent thickets, and royalty stacking were of practical relevance [2]. The NRC (National Research Council) provided a series of case studies on the use of patents, which covered a small number of important research tools (especially foundational upstream discoveries), and found that "restricted access" to upstream discoveries and tools had occurred [5]. Murray and Stern constructed a set of 169 patent-paper pairs from the US and a control group comprising non-patent-paper pairs. The pattern of forward citations to scientific articles of the patent-paper pairs was then compared with that of the control group. On the basis of this analysis, it was shown that after the patent grant was issued, the citation rate of the related paper declined by between 9% and 17%, indicating that a modest anticommons effect occurred [9].

Walsh et al. conducted 70 interviews with several different innovation entities (ie, pharmaceutical firms, biotech firms, university researchers, technology transfer officers, patent lawyers, etc). In contrast to other studies, they observed less breakdown or restricted access to research tools than expected [7]. They argued that some related problems (for example, royalty stacking, expensive licensing fees for tools, etc.) were manageable and that the benefits were larger than the costs. Nicol and Nielsen (2003) conducted a survey and a number of interviews in Australia, and the participants reported that they had rarely experienced difficulties in accessing broadly applicable research tools and technologies [11]. In an IPI (Intellectual Property Institute) study carried out in the UK, respondents answered that the patent thicket had not materialized and that "they did not feel that genetic sequence patents had had a negative impact on R&D" [12]. Resnik (2001) examined the current climate of DNA patents and concluded that the benefits outweigh the drawbacks [13]. According to an AAAS (American Association for the Advancement of Science) survey, only 11% of Japanese scientists reported some difficulties in acquiring patented technology in the past five years [15]. Furthermore, Azoulay et al. examined the effects of the patenting behavior of academic life scientists in a panel dataset and found that both the flow and stock of the scientists' patents were positively related to their subsequent publication record. The positive correlation between patent applications and the flow of publications suggests that patents and papers encode similar pieces of knowledge, and patents do not crowd out the level of scientific publications [16,17].

In summary, the effects of patents on subsequent innovations in the field of biotechnology are still unclear in developed countries. It is important to investigate the effects of prior patents on innovation in developing countries because innovations in these countries are usually incremental or follow-on innovations. In this study, the effects of patents, especially research tool patents (see Note B), on innovation were examined using survey data and statistical analysis.

## Methods

A survey of researchers in the biotechnology industry was conducted between March and April 2008. The sample frame for the survey comprised senior researchers in biotech SMEs (small- and medium-sized entrepreneurs) that were listed in the book "Bio-venture 2007" published by the Korea Bio Venture Association (KOBIOVEN). The survey comprised a number of questions with respect to the effects of upstream discoveries and patented research tools, including whether the respondents have used patented research tools, how they have acquired patented research tools, whether they experienced difficulties in acquiring patented research tools, what are the major causes of these difficulties, and what is the main effect of patents (see Appendix A). The data was analyzed using statistical methods to assess the researchers' experience in using research tools and the effects of research tool patents.

### Hypothesis

Research question 1: **Concerns: Where do they occur?**

This question was posed to determine whether the concerns were more widespread in the biomedical sector than in other sectors. Many previous studies have only investigated the effects of patents on the biotechnology industry [7-13], particularly the biomedical sector. If researchers in the biomedical sector felt a higher level of anxiety with regard to patents, it would be valid to examine the effects of patents primarily in this sector. Hence, it was proposed that

*Hypothesis 1. Researchers who work in the biomedical sector view the patent system more negatively than researchers in other sectors*.

Research question 2: **Does it deter innovation?**

Some scholars have surveyed whether research projects had been changed or abandoned because of difficulties with prior patents [7,14,15] to determine if "restriction is a matter of degree." This type of inquiry was a kind of direct measurement. In this study, we used not only a direct measurement but also other indirect measurements such as "attitudes toward patents" and "levels of innovation performance."

If restricted access problems were serious, they would affect researchers' attitude toward patents. Hence, it was supposed that

*Hypothesis 2. Researchers who experienced difficulties with prior patents evaluated patents (effects of patents) more negatively*.

Indeed, if the restricted access problems were serious, they would negatively affect the levels of innovation performance. Hence, it was supposed that

*Hypothesis 3. Experiencing difficulties with prior patents and levels of innovation performance were negatively correlated*.

### Variables

Participants were asked to choose "the main effect of patents on innovation" from a list that comprised positive or negative terms (positive terms: fostering information sharing, improving productivity; negative terms: increase in cost, restrictions on access to information, stifling effect on subsequent innovation). The response terms were then converted into two categories and given a score of -1 for a negative term and +1 for a positive term. In addition, participants were asked to evaluate "the effects of patents on innovation." The response scores were then converted to five response categories and given a score between -2 and +2 (negative to positive). To determine where the concerns occurred, the scores of two groups (the biomedical sector vs. other sectors) were compared with each other using a t-test analysis (see Appendix B). Details of the compared values are given in Table 1.

**Table 1:** Variables (X) considered in t-test analysis

Variable	Score		Definition
Main effect of patents	+1	(positive terms)	Fostering information sharing, improving productivity
	
	--1	(negative terms)	Increase in costs, restrictions on access to information, stifling effect on subsequent innovation

Evaluating effects of patents on innovation	--2 ~ +2		--2 = highly negative; --1 = negative; 0 = neither negative nor positive; +1 = positive; +2 = highly positive

If the restricted access problems were serious, they would affect the researchers' attitude toward patents. To test whether the restricted access problems were significant, "the degree of difficulty" in acquiring research tools was measured by five response categories and given a score of 1 to 5 (very easy to very difficult). Thereafter, the scores representing "the degree of difficulty" were regressed on "the effects of patents for innovation" using OLR (ordered logit regression) (see Appendix B). In this study, corporate size, proxied by the log number of employees, and corporate age were used as the control variables. Details of these variables are given in Table 2.

**Table 2:** Variables (X) considered in OLR analysis

Classification	Variable	Definition
Independent variable	Attitude toward patents (Evaluating effects of patents for innovation)	--2 = highly negative; --1 = negative; 0 = neither negative nor positive; +1 = positive; +2 = highly positive

Dependent variable	Degree of difficulty in acquiring research tools	1 = very easy; 2 = easy; 3 = neither easy nor difficult; 4 = difficult; 5 = very difficult

Control variable	Size	Log number of employees in 2007
	
	Age	Number of months until 2007

If the restricted access problems were serious, they would affect the level of innovation performance. Poisson regression (see Appendix B) was performed to test the relationship between a firm's innovation performance (proxied by the total number of patents) and independent variables that included "the degree of difficulty" in acquiring research tool patents and two control variables. In this study, corporate size, proxied by log number of employees, and corporate age were used as the control variables. Details of these variables are given in Table 3.

**Table 3:** Variables (X) considered in Poisson regression

Classification	Variable	Definition
Independent variable	Innovation performance	Total number of patents

Dependent variable	Degree of difficulty in acquiring research tools	1 = very easy; 2 = easy; 3 = neither easy nor difficult; 4 = difficult; 5 = very difficult

Control variable	Size	Log number of employees in 2007
	
	Age	Number of months until 2007

## Results and discussion

A total of 109 responses were received (specifically, 39 from the biomedical sector, 36 from the bio-food sector, 15 from the biochemical sector, and 19 from other sectors), which resulted in a response rate of 33.4% (109/326, see Appendix C).

### 1. Experience in the Korean biotech SMEs

#### Use of research tools

**Figure 1:**
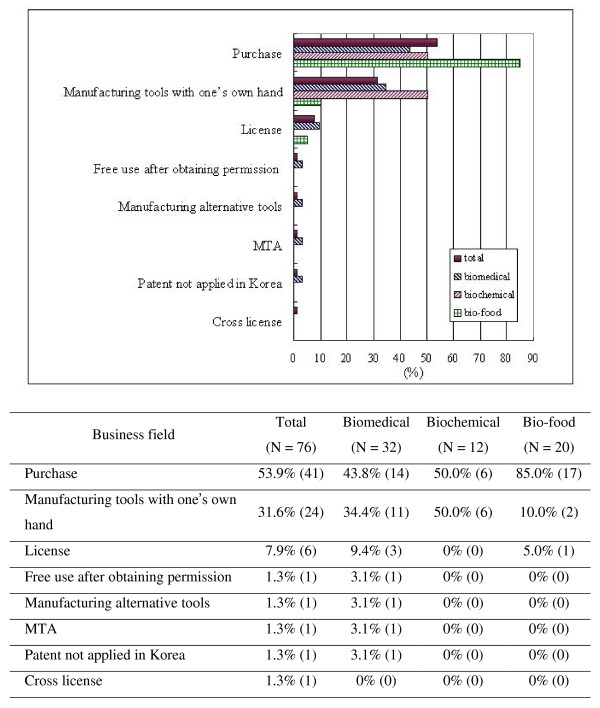
**Methods for acquiring patented research tools**.

In the survey, the participants were asked whether they had used research tools in their work; 69.7% (76/109) of the respondents reported that they had done so. As depicted in Figure 1, most of the respondents who had used patented research tools acquired them by purchasing (53.9%, 41/76), and this method was used most frequently in the bio-food sector (85.0%, 17/20). In addition, 31.6% (24/76) of the respondents who had used patented research tools reported that they had made their own tools. This result was similar to the findings of another study conducted by Walsh et al. Walsh et al. found that "one-third of the industrial respondents acknowledged occasionally using patented research tools without a license. The firms felt that much of their research would not yield commercially valuable discoveries, and thus, they saw little need to spend money to secure the rights to use the input technology" [7]. In Korea, there is a statutory research exemption in patent law (see Note C), and most of the respondents believed that this research exemption should be applied more broadly. The KIPO (Korean Industrial Property Office) has not yet clearly defined the scope of "research exemption," but it may be possible to interpret it more narrowly than what researchers have invoked. In this case, using patented technologies without permission is considered as an infringement; thus, there exist other additional potential problems.

#### Difficulties in acquiring patented research tools

Participants were asked whether they had experienced difficulties in acquiring patented research tools. In this survey, 21.1% (16/76) of the respondents who had used research tools reported that they had experienced some difficulties. This was particularly true for researchers in the biomedical sector, who frequently experienced difficulties in acquiring patented research tools (31.3%, 10/32).

**Figure 2:**
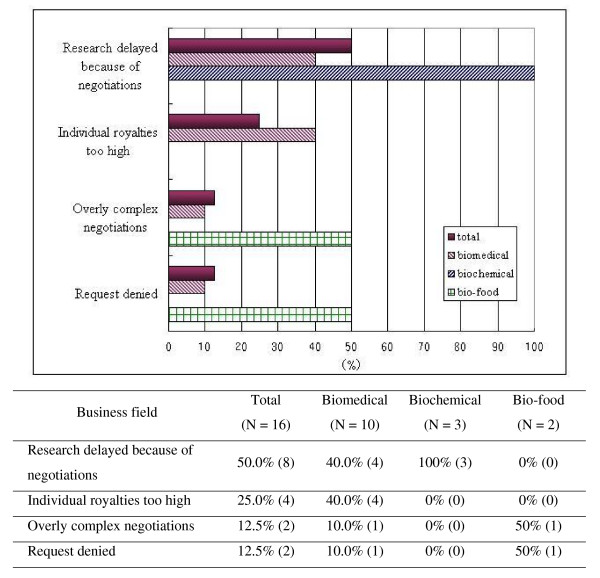
**Causes of difficulties in using patented research tools**.

The most significant issue observed in this study was that the progress of research was delayed because of the length of negotiations (50% of the respondents experienced difficulties, 8/16). Twenty-five percent (4/16) of the respondents indicated that individual royalties were too high, while some of the respondents (12.5% of the respondents experienced difficulties, 2/16) reported that they were denied the use of patented research tools by patent owners. In addition, some of the respondents (12.5% of the respondents experienced difficulties, 2/16) reported that the negotiations were overly complex (see Figure 2).

Indeed, 17.1% (13/76) of the respondents who had used research tools reported that they had to abandon their projects due to research tool (or upstream discovery) patents.

These results provided some evidence on the existence of restricted access problems, even though the frequency was not high. Furthermore, if a broad scope of the "research exemption" is not allowed, restricted access problems will occur more frequently.

### 2. Concerns: Where do they occur?

Participants were asked to choose "the main effect of patents on innovation" and evaluate "the effects of patents on innovation." To determine *where concerns occur*, the scores of two groups (the biomedical sector vs. other sectors) were compared with each other using a t-test analysis.

The results of this analysis suggest that there were statistically significant differences between the biomedical sector and other sectors. As depicted in Table 4, researchers in the biomedical field evaluated patents more negatively. It appears that the concerns were more widespread in the biomedical sector than in other sectors, because follow-on innovations rely more heavily on prior scientific findings in this sector than in other sectors; moreover, ethical and moral issues are associated with patents. These results indicate that it was valid to conduct these types of studies primarily in the biomedical sector.

**Table 4:** Where do concerns occur? t-test analysis

	Business field	N	Mean	Std. deviation	t	Df	Sig. (2-tailed)
Main Effect***	Biomedical	39	--0.026	1.013	--2.975	67.652	0.004
				
	Others	70	0.543	0.846			

Evaluating effects**	Biomedical	39	--0.282	0.972	--2.483	107.000	0.015
				
	Others	70	0.143	0.785			

*** denotes statistical significance at the < 0.01 level.

** denotes statistical significance at the < 0.05 level.

### 3. Does it deter innovation? The current situation

If restricted access problems were serious, they would affect the researchers' attitude toward patents. To test whether the restricted access problems were significant, the scores representing "the degree of difficulty" were regressed on "attitude toward patents–the effects of patents for innovation" using OLR. As depicted in Tables 5 and 6, the results obtained from the biotechnology field were statistically insignificant; however, attitudes toward patents (evaluating effects of patents) and experiencing difficulties with prior patents were positively correlated in the biomedical sector (β = 0.511, p < 0.10). Researchers in this sector believed that patents had a positive effect on subsequent innovations (by fostering R&D investment and information sharing among researchers and improving research productivity). This indicates that the benefits of patents are larger than the costs, and the restricted access problems are not significant. This result is consistent with the results of previous studies [7,10-13].

**Table 5:** Are "restricted access problems" serious in the biotechnology industry? OLR results (1)

Evaluating effects	Coeff.	Std. err.	P > z
Difficulty	0.121	0.189	0.521

Size	0.188	0.475	0.693

Age**	0.014	0.006	0.031

	Number of obs.	76	
	Log likelihood	--90.164	
	LR chi2(3)	6.75	
	Prob > chi2	0.080	

** denotes statistical significance at the < 0.05 level.

**Table 6:** Are "restricted access problems" serious in the biomedical sector? OLR results (2)

Evaluating effects	Coeff.	Std. err.	P > z
Difficulty*	0.511	0.302	0.091

Size	--0.151	0.646	0.815

Age**	0.021	0.010	0.038

	Number of obs.	32	
	Log likelihood	--39.123	
	LR chi2(3)	7.32	
	Prob > chi2	0.063	

** denotes statistical significance at the < 0.05 level.

* denotes statistical significance at the < 0.10 level.

In addition, if the restricted access problems were serious, they would adversely affect innovation performance. Poisson regressions were performed to test the relationship between a firm's innovation performance (proxied by the total number of patents) and independent variables that included "the degree of difficulty" in acquiring research tool patents and two control variables. Descriptive statistics for variables are given in Table 7. As detailed in Table 8, the degree of difficulty was positively associated with corporate innovation performance (β = 0.073, p < 0.05) in the biotechnology industry. In the biomedical sector, the correlation coefficient between the degree of difficulty and level of innovation performance was positive, but the result was statistically insignificant (β = 0.116, p = 0.200; see Table 9). Therefore, we concluded that the difficulties in acquiring patented research tools have not yet seriously deterred innovation. This result is consistent with the results of previous studies [7,10-13].

**Table 7:** Descriptive statistics and correlation (n = 76)

	Mean	S. D.	Difficulty	Size	Age
Difficulty	2.566	1.159	1.000		

Size	1.276	0.473	0.030	1.000	

Age	95.658	38.037	--0.091	0.357	1.000

**Table 8:** Restricted access problems and innovation performance in the biotechnology industry: Poisson regression results of variables versus innovation performance

Innovation performance	Coeff.	Std. err.	P > z
Constant***	1.377	0.266	0.000

Difficulty**	0.073	0.034	0.033

Size*	0.290	0.171	0.090

Age	0.003	0.002	0.242

Sigma	0.826	0.048	0.000

	Number of obs.	76	
	Log likelihood	--856.413	
	Chi squared	1121.803	
	Prob > chi2	0.000	

Poisson model with normal heterogeneity

*** denotes the correlation coefficient observed at significance level < 0.01.

** denotes the correlation coefficient observed at significance level < 0.05.

* denotes the correlation coefficient observed at significance level < 0.10.

**Table 9:** Restricted access problems and innovation performance in the biomedical sector: Poisson regression results of variables versus innovation performance

Innovation performance	Coeff.	Std. err.	P > z
Constant**	1.289	0.612	0.035

Difficulty	0.116	0.091	0.200

Size	0.156	0.352	0.657

Age	0.004	0.006	0.512

Sigma	0.862	0.094	0.000

	Number of obs.	32	
	Log likelihood	--128.999	
	Chi squared	678.769	
	Prob > chi2	0.000	

Poisson model with normal heterogeneity

** denotes the correlation coefficient observed at significance level < 0.05.

## Conclusions and recommendations for future research

Based on the study of the impacts of patents on innovation in the Korean Biotech SMEs and a comparison of these results with previous studies, we conclude that although restricted access problems have occurred, this has not yet deterred innovation in Korea. However, we can state that potential problems do exist, and the effects of restricted access should be constantly scrutinized.

This study has some limitations that should be accounted for in future studies. The biotechnology industry is an emerging field whose development largely relies on research-intensive SMEs; thus, in this study, we focused on biotech SMEs [18]. However, there are other actors of innovation, for example, universities, public research institutes, and pharmaceutical firms. Further analysis should include these actors.

### Note A: Instance

"For instance, any scientist who wants to study the genetics of breast cancer needs to utilize the BRCA 1 test." [10]

### Note B: Definition of research tools

The NIH (National Institutes of Health) defines research tools as "embracing the full range of tools that scientists use in the laboratory." According to the OECD (Organization for Economic Cooperation and Development), "research tools may be considered compositions or methods used in conducting experiments. This term could embrace a broad range of resources that scientists use in the laboratory including, but not limited to, cell lines, monoclonal antibodies, reagents, animal models, growth factors, combinational chemistry, genomic and proteomic libraries, drug and drug targets, clones and cloning tools, methods, laboratory equipment and machines, databases and software."

### Note C: Related section

"The effects of the patent right shall not extend to the following: (i) working of the patented invention for the purpose of research or experiment..." (Section 96-(1) of the Patent Law in Korea).

## Authors' contributions

KK participated in the design of the survey study, performed the statistical analysis, and drafted the manuscript. TR also participated in the design of the survey. YL conceived the study and helped in drafting the manuscript. All authors read and approved the final manuscript.

## Appendix A: Questionnaire

Q1. What is the main business field of your company?

1) Biomedical 2) Biochemical 3) Bio-food 4) Bio-environment

5) Bio-energy 6) Bio-electronics 7) Bio-process 8) Bioinformatics

Research tools embrace the full range of tools that scientists use in the laboratory. This term includes, but not limited to, cell lines, monoclonal antibodies, reagents, animal models, growth factors, combinational chemistry, genomic and proteomic libraries, drug and drug targets, clones and cloning tools, methods, laboratory equipment and machines, databases, and software. Patented research tools are research tools protected by patent law.

Q2. Have you used any patented research tools in your research? (Please answer within the context of your employment.)

1) Yes 2) No

Q3. Which of the following methods was mainly used in the acquisition of the patented research tools?

1) Manufacturing tools with one's own hand

2) Manufacturing alternative tools

3) Purchase

4) Free use after obtaining permission

5) License

6) Cross-license

7) Material Transfer Agreement (MTA)

8) Free use because the patent(s) is (are) not applied in Korea

9) Other ___________________________________

Q4. How easy or difficult was it to acquire the patented research tools?

1) Very easy 2) Easy 3) Neither easy nor difficult

4) Difficult 5) Very difficult

Q5. If you had experienced difficulties, what was the main reason?

1) Research was delayed because of negotiations

2) Individual royalties were too high

3) Overly complex negotiations

4) Royalty stacking

5) Licensing negotiations broke down

6) Requests denied

7) Unable to determine the patent status of the research tool

8) Other ____________________________________

Q6. Has your research been changed due to patented research tools or prior upstream discoveries?

1) Yes 2) No

Q7. Has your research been abandoned due to patented research tools or prior upstream discoveries?

1) Yes 2) No

Q8. What do you think is the main effect of patented research tools or prior upstream discoveries?

1) Fostering information sharing among researchers

2) Improving research productivity

3) Increasing research cost

4) Restricting access to information

5) Stifling effect on subsequent innovation

6) Other ________________________________

Q9. Please evaluate the effects of patents on innovation

1) Positive effect: _______ % + 2) Negative effect: _______ % = 100%

Q10. Has your company applied for patents? 1) Yes 2) No

If yes, how many patents have been applied for? _______________

## Appendix B: Statistical models

An independent t-test analysis was used to investigate hypothesis 1 by comparing the differences in attitudes of scientists in biomedical and other fields toward the patent system. The t-test was used to compare the values of the means from two samples and to test whether it is likely that the samples are from populations having different mean values [19]. The formula for the independent t-test is



where X_1 _denotes the mean for group 1; X_2_, the mean for group 2; SS_1_, the sum of squares for group 1; SS_2_, the sum of squares for group 2; n_1_, the number of subjects in group 1; and n_2_, the number of subjects in group 2.

An OLR model and Poisson regression model were used to investigate hypotheses 2 and 3: **Does it deter innovation? The current situation**.

The OLR is used when the variables are ordinal dependent variables [20]. The ordered logit model has the following form:



The data for the number of scientific publications falls in the category of count data. The Poisson regression model has been widely used to study such data [21]. The primary equation of this model is



The most common formulation for λ_i _is the log-linear model,

ln *λ*_*i *_= *β'X*_*i*_

The variables were regressed on a set of factors integrated into the equation.

## Appendix C: A note on the interpretation of statistics

The response rate for the survey was 34.4%. The low r esponse rate could have caused unmeasured bias in the statistics given in this manuscript. To address this issue, the main characteristics of the companies that responded to the survey were compared with those of the companies that did not respond to determine if our results represent a biased subset. All the companies were listed in the book "Bio-venture 2007" published by KOBIOVEN.

On the basis of this analysis, we found that the companies that responded and the companies that did not respond had a similar age, numbers of employees and patents, and similar distributions in the business field. These results indicate that the low response rate of our survey did not induce a bias (see Table 10).

**Table 10:** Comparing respondents and nonrespondents

Main Characteristics	Respondents	Nonrespondents	Sig.
Age	7.9 yr	7.3 yr	n.s.
Size (number of employees)	30.7	23.8	n.s.
Number of patents	9.0	8.4	n.s.
Business fields			
Biomedical field	35.8%	28.6%	n.s.
Biochemical field	13.8%	15.7%	n.s.
Bio-food field	33.0%	23.5%	n.s.

Note: Respondents, n = 109; nonrespondents, n = 217; data for 2006
